# The time-course of a scrapie outbreak

**DOI:** 10.1186/1746-6148-2-20

**Published:** 2006-06-13

**Authors:** K Marie McIntyre, Simon Gubbins, Wilfred Goldmann, Emily Stevenson, Matthew Baylis

**Affiliations:** 1 Institute for Animal Health, Pirbright Laboratory, Ash Road, Pirbright, Surrey, UK; 2 Institute for Animal Health, Neuropathogenesis Unit, Ogston Building, West Mains Road, Edinburgh, UK; 3 University of Liverpool Veterinary Teaching Hospitals, Leahurst, Chester High Road, Neston, South Wirral, UK

## Abstract

**Background:**

Because the incubation period of scrapie has a strong host genetic component and a dose-response relationship, it is possible that changes will occur during an outbreak, especially in the genotypes of cases, age-at-onset of disease and, perhaps, the clinical signs displayed. We investigated these factors for a large outbreak of natural scrapie, which yielded sufficient data to detect temporal trends.

**Results:**

Cases occurred mostly in two genotypes, VRQ/VRQ and VRQ/ARQ, with those early in the outbreak more likely to be of the VRQ/VRQ genotype. As the epidemic progressed, the age-at-onset of disease increased, which reflected changes in the genotypes of cases rather than changes in the age-at-onset within genotypes. Clinical signs of cases changed over the course of the outbreak. As the epidemic progressed VRQ/VRQ and VRQ/ARQ sheep were more likely to be reported with behavioural changes, while VRQ/VRQ sheep only were less likely to be reported with loss of condition.

**Conclusion:**

This study of one of the largest scrapie outbreaks in the UK allowed investigation of the effect of PrP genotype on other epidemiological parameters. Our analysis indicated that, although age-at-onset and clinical signs changed over time, the observed changes were largely, but not exclusively, driven by the time course of the PrP genotypes of cases.

## Background

Scrapie is an infectious disease, but one that is unusual in that the incubation period leading to clinical disease is governed by a strong host genetic component [1] and a strong dose-response relationship [2]. These factors can interact to yield intriguing epidemiological patterns.

During an outbreak, the PrP genotypes of affected animals are expected to change over time. Those encoding PrP genotypes with the shortest incubation periods should be the first to reach clinical onset, followed by their co-occurrence with those encoding PrP genotypes with longer incubation periods, then those with the longest incubation periods. If most sheep are infected at a similar age, this could manifest as a change in the age-at-onset of scrapie over time. The level of infectivity to which susceptible animals are exposed could also increase through the epidemic as the cumulative total of infected animals rises. A higher dose may shorten the incubation period and lead to a reduction in the age-at-onset over time. If PrP genotype, incubation period or age-at-onset change with time during an outbreak, then any linkage between any of these factors and the clinical signs of scrapie could also lead to changes in the clinical manifestation of the disease over time. A time course of clinical signs may also arise from the farmer, who may be able to recognise scrapie on the basis of fewer or different clinical signs as more cases are encountered.

These ideas are supported by the reports of farmers running commercial flocks affected by natural scrapie. In a field study undertaken by the Institute for Animal Health (IAH), twenty-four percent of farmers reported a change in the clinical signs being reported through the course of a scrapie epidemic, while thirty-five percent reported a change in the ages of cases.

Detection of a time trend, and teasing apart the causal factors behind it, is made difficult by the small number of cases confirmed in most outbreaks and the range of PrP genotypes involved. Furthermore, variability between flocks in the characteristics of outbreaks precludes, in most cases, the combining of datasets.

Here, we have investigated in detail the scrapie outbreak of one of very few flocks for which sufficient data are available for time course analysis. Between 1997 and 2004 there were over 130 confirmed cases of scrapie in a single flock of Welsh Mountain sheep. Almost all cases were in just two genotypes. This outbreak is possibly the largest reported for any flock in Great Britain over the same time period. The scale of the outbreak gave us an opportunity to explore in detail the time course of the scrapie epidemic at the level of PrP genotype and with extensive detail of the clinical presentation of disease.

## Results

### The flock

Ten (out of 15) PrP genotypes were found in this flock upon blood sampling in 2001 (Table 1). A quarter (25.2%) carried the VRQ allele (associated with the highest risk of scrapie), but only 0.5% were of the most susceptible genotype, VRQ/VRQ. The genotype frequencies correspond to allele frequencies of: ARR, 39.9%; AHQ, 14.7%; ARQ, 32.2%; and VRQ, 13.2%. The ARH allele was not detected in this flock. Animals also had PrP haplotypes ARQ-F^141 ^and ARQ-S^241 ^which occurred at frequencies of 21.5% and 14.2%, respectively. Neither of these haplotypes was associated with the occurrence of scrapie in this flock.

### The outbreak

The farmer did not know at what point his flock acquired infection. Although he thought that there had been one case of scrapie (unconfirmed) in 1982, he did not recognise any more animals with scrapie-like signs until the start of the current epidemic in September 1997. The affected animals exhibited clinical signs of incoordination (ataxia), nervousness/excitability (nervous) and rubbing/scratching (pruritis); he thought that both the clinical signs and the age of the animals succumbing to disease changed during the course of the epidemic.

**Figure 1:**
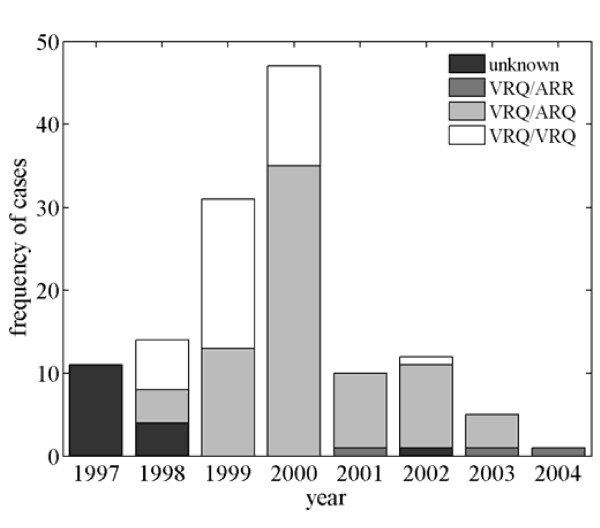
Scrapie epidemic in a flock of Welsh Mountain sheep: frequency of confirmed cases by PrP genotype.

Between September 1997 and February 2004 there were 131 confirmed cases in the flock, in animals of 3 genotypes (Table 1). The frequency of cases increased to a peak in 2000 and subsequently decreased (Figure 1). The number of cases in 2001 appears unusually low; this may be related to difficulties in reporting scrapie during the UK's foot-and-mouth disease epidemic. More cases were reported in the first than the second half of each year, but seasonal trends were not significant (*t*_13 _= 0.54, *P *= 0.59).

Most cases were initially in animals of the VRQ/VRQ genotype, which peaked in 1999, after which cases became more prevalent in animals of the VRQ/ARQ genotype, which peaked in 2000 (Figure 1). Three cases also occurred in animals of the less-susceptible VRQ/ARR genotype during the decline phase of the epidemic (Figure 1). Considering only the time from when the flock was blood sampled, the proportion of animals which subsequently died from scrapie were: 2.1% (2/96) in VRQ/ARR; 27.0% (17/63) in VRQ/ARQ; and 25.0% (1/4) in VRQ/VRQ.

### The time-course

**Figure 2:**
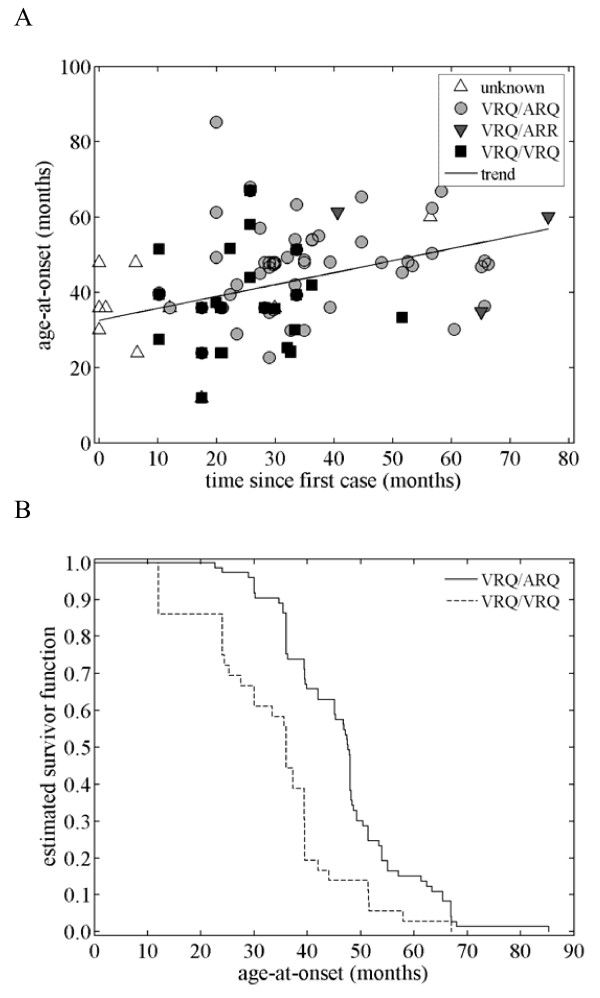
**Age at the onset of clinical signs**. (A) Age at onset and its dependence on genotype and time since the first case in the outbreak. The observed age at onset is shown for each genotype: VRQ/VRQ (black squares), VRQ/ARQ (grey circles), VRQ/ARR (grey downward triangles) and unknown (white upward triangles). A fitted trend line is also shown. (B) Kaplan-Meier estimates of the survivor function for the age at onset (in months) in VRQ/VRQ and VRQ/ARQ animals.

As predicted, the genotype of cases changed during the time-course of the outbreak (Figure 2A). Earlier in the epidemic cases were more likely to be of the VRQ/VRQ than the VRQ/ARQ genotype (AOR = 0.93; 95% CI: 0.90–0.98). Exploratory analysis suggested that animals of the VRQ/ARR genotype were likely to exhibit clinical signs later in the epidemic than either VRQ/ARQ or VRQ/VRQ animals (Figure 2A), but their small sample size precluded more detailed analyses.

As observed by the farmer, cases which occurred later in the epidemic tended to be older than those seen earlier (Figure 2A; *F*_1,129 _= 28.951, *P *< 0.001). Cases of the VRQ/VRQ genotype developed clinical signs at an earlier age than VRQ/ARQ animals (Figure 2B; log-rank test: χ^2 ^= 16.90, df = 1, *P *< 0.001), but age within each genotype did not change significantly during the outbreak (*P *= 0.329). This implies that the increase in age-at-onset was due to changes in the PrP genotype of cases rather than changes in age within each genotype.

### Clinical signs

Analysis was undertaken for those clinical signs with sufficient observations (n>25): ataxia, change of behaviour, fleece loss, loss of condition, pruritus and trembling. As the epidemic progressed, animals were more likely to be reported showing a change in behaviour, independent of PrP genotype (AOR = 1.09; 95% CI: 1.05–1.13). VRQ/VRQ animals were less likely to show loss of condition (AOR = 0.77; 95% CI: 0.62–0.97) or fleece loss (AOR = 0.83; 95% CI: 0.72–0.94) as the outbreak progressed. This pattern was not seen in VRQ/ARQ cases. Throughout the outbreak, older cases of any genotype were more likely to show ataxia (AOR = 1.03; 95% CI: 1.00–1.06) and trembling (AOR = 1.06; 95% CI: 1.02–1.09) than younger ones. Finally, animals exhibiting fleece loss were more likely to be of the VRQ/VRQ rather than the VRQ/ARQ genotype (AOR = 3.29; 95% CI: 1.21–8.95).

## Discussion

Although there have been several studies describing within-flock outbreaks of scrapie [3-7] the results of this paper represent the first analysis addressing confounding between the effects of time since the beginning of the epidemic, age at onset and PrP genotype. The major limitations of this study are that we do not know when the flock first became infected with scrapie; and genotypes were unavailable for 16 cases, including the first 14 during the epidemic.

An interesting feature of this outbreak is that cases occurred in the VRQ/VRQ, VRQ/ARQ and VRQ/ARR genotypes only, despite the high frequency of sheep of the ARQ/ARQ and VRQ/AHQ genotypes in the flock (Table 1). These findings confirm the resistance of the VRQ/AHQ genotype to classical scrapie [8,9] and the resistance of the ARQ/ARQ genotype to certain scrapie strains in some sheep breeds [10,11].

A key finding of this study is that the cases which occurred later in the outbreak tended to be older. Our analysis has shown that this is not related to changes in the age-at-onset within each genotype during the outbreak. Rather, it is an effect of the PrP genotype of cases, which influences the age at which animals succumb to disease. Our results differ from an earlier study, which reported a decline in the age-at-onset for four outbreaks [7], though the decline was significant (P < 0.001) in only one outbreak in a flock of Suffolk sheep [4]. This Suffolk flock was bred to maximise the incidence of disease; hence, susceptible animals were constantly being introduced to the flock and exposed to an increasing load of infection. By contrast, most, though not all, susceptible animals in our study flock succumbed to scrapie, but their susceptible alleles were not replaced. The resulting pattern of the outbreak means that animals of the same genotype were, on average, exposed to similar loads of infection.

Animals of the VRQ/VRQ genotype had a younger age-at-onset than VRQ/ARQ animals (Figure 2), in common with other studies [3,8,10]. The age at onset of clinical signs depends on a number of factors, including the infectious load, age-dependent exposure, age-dependent susceptibility and incubation period. Modelling analysis of an outbreak in a Cheviot flock suggested that the incubation period for VRQ/VRQ animals was shorter than that for VRQ/ARQ and that there was evidence for age-dependent susceptibility [12]. This suggests that the differences in the age-at-onset are likely to reflect differences in incubation period rather than age-dependent exposure. More detailed analyses are needed, however, to tease apart the interactions of various age and genotype effects in the age-at-onset of clinical signs.

Our study is the first to report changes in clinical signs during a scrapie outbreak. Animals were more likely to be reported showing a change of behaviour as the epidemic progressed, irrespective of PrP genotype. One hypothesis would be that at the start of the epidemic the farmer required physical signs to identify scrapie, but as the epidemic progressed he became better able to detect disease on the basis of more subtle behavioural changes. Indeed, behavioural changes are reported prior to the onset of other clinical signs in both goats [13] and mice [14].

Temporal changes in clinical signs also differed between PrP genotypes. Early in the epidemic, VRQ/VRQ animals were more likely to show loss of condition and fleece loss/change, while those reported later in the epidemic were less likely to do so. This could be related to the aforementioned greater reliance of the farmer on physical (as opposed to behavioural) signs earlier in the outbreak; however, this seems unlikely, as the pattern did not occur in animals of the VRQ/ARQ genotype. Alternatively the effect may be real, or it may reflect variation in the clinical signs recorded by the seven reporting officers. Exploratory analysis provided some support for this latter hypothesis, but there were too few cases to be able to test this rigorously.

## Conclusion

In this paper we have described one of the largest scrapie outbreaks in the UK. As the cases were almost exclusively in two genotypes, this has facilitated the investigation of the effect of PrP genotype on other epidemiological parameters. Although age-at-onset and clinical signs changed over time, our analysis indicated that the effect was largely, but not exclusively, driven by the time course in the PrP genotype of cases.

## Methods

### Flock details

A flock comprising sheep predominantly of the Welsh Mountain breed and numbering about 850 breeding animals was recruited into the aforementioned IAH scrapie field study. The first cases of scrapie within this flock were confirmed in September 1997. As of February 2004, there were 131 confirmed cases of scrapie. The entire breeding flock (n = 829) was blood sampled for PrP genotyping in August 2001.

### Identification of confirmed cases

The data for scrapie cases within the study flock were retrieved from the Scrapie Notification Database (SND) held at the Veterinary Laboratories Agency (VLA). This includes the breed, sex, date of birth, date of death, PrP genotype and clinical signs in animals submitted as suspect scrapie cases. Tissue samples are also obtained from cases, which are subject to routine analysis for evidence of scrapie.

### Breed of cases

The confirmed cases in the flock were a mixture of pure-bred (n = 117) and cross-bred Welsh Mountain (n = 4), Welsh Half-bred (n = 3) and Beulah Speckled Face (n = 7). Because of the small number of cases in most breeds and crossbreeds within the flock, breed was not considered further in the analyses.

### PrP genotype analysis

Susceptibility to scrapie is strongly associated with polymorphisms in the prion protein (PrP) gene [8,9,15], of which five alleles are commonly found (defined by amino acids at codons 136, 154 and 171): VRQ, ARQ, ARH, AHQ and ARR [10,16]. PrP genotype analysis was undertaken using approximately 5 ml of blood collected into an EDTA-vacutainer from each sheep, as described previously [17].

### Clinical signs

The clinical signs used within analyses were those observed and recorded by the reporting officers who visited the farm when notified by the farmer of a suspect case. Data on clinical signs were available for 120 out of 131 confirmed cases; recorded signs included abnormal head position, ataxia, change of behaviour (or temperament), biting, dribbling (of saliva or cud), fleece loss (or change), loss of condition, nervousness, nibbling reflex, pruritus, teeth grinding, trembling and visual impairment.

### Statistical methods

'Age' in months relates to when clinical signs were reported; 'time' in months was from the first confirmed scrapie case (i.e. the start of the reported epidemic). Analyses were restricted to confirmed clinical cases of scrapie.

A binary logistic regression model was used to examine the effects of time and age upon the PrP genotype of cases. Cox proportional hazard models, with age as the survival measure, were used to analyse the effect of time and PrP genotype upon age [18]. Binary logistic regression models were used to investigate the effect of time, age and PrP genotype on the presence or absence of clinical signs. Statistical significance was determined by a *P*-value of less than 0.05. Models were derived by step-wise deletion of insignificant main effects, excluding those within significant interaction terms. Within logistic regression models, the adjusted odds ratios (AOR) and 95% confidence intervals (CI) were calculated for significant independent variables using standard methods.

## Authors' contributions

KMM carried out the statistical analyses and drafted the manuscript. SG carried out the statistical analyses and critically revised the manuscript. WG and ES undertook the PrP genotyping. MB conceived the study and critically revised the manuscript. All authors read and approved the final manuscript.

## Figures and Tables

**Table 1:** PrP genotype frequencies in the breeding flock of 829 sheep in August 2001, and in confirmed cases between 1997 and 2004.

PrP genotype	Animals in flock in 2001		Cases 1997–2004	
	
	Number	%	Number	%
ARR/ARR	124	15.0	-	-
ARR/AHQ	80	9.7	-	-
ARR/ARQ	220	26.5	-	-
AHQ/AHQ	17	2.1	-	-
AHQ/ARQ	77	9.4	-	-
ARQ/ARQ	80	9.5	-	-
VRQ/ARR	96	11.6	3	2.3
VRQ/AHQ	46	5.5	-	-
VRQ/ARQ	63	7.6	75	57.3
VRQ/VRQ	4	0.5	37	28.0
Unknown	22	2.7	16	12.2
